# ABCDE: Directing Student Observation During High-Fidelity Simulation

**DOI:** 10.1007/s40670-020-01081-1

**Published:** 2020-09-24

**Authors:** Josephine Seale, Abubakar Khan, Barnaby Hirons, Colin Butchers

**Affiliations:** 1 GKT School of Medical Education, Simulation and Interactive Learning Centre (SaIL), King’s College London, Shepherds House, London, UK; 2 Postgraduate Medical Department, Weston Education Centre, King’s College Hospital, London, UK

**Keywords:** High-fidelity simulation, Medical undergraduate, Observation tool, Active learning

## Abstract

To encourage an active learning environment during the observation stage of high-fidelity simulation, an observation tool was created to help students recognise and record the technical and non-technical skills identified whilst watching their peers. Future work will involve quantifying any educational benefits of the tool across the medical student cohort.

## Innovation

Simulation-based education has become an established means of enabling medical students to both learn and practise technical and non-technical skills in a safe environment. As a result of large student numbers, the use of simulation in an undergraduate setting typically entails students alternating between participating and observing roles.

High-fidelity simulation (Hi-Fi SIM) has been incorporated throughout the undergraduate medical curriculum at King’s College London (KCL). The aims of these sessions are two-fold: to enhance student knowledge and appreciation of the use of technical and non-technical (human factors) skills in clinical situations and to provide students the opportunity to develop and practise these skills in a low-risk environment. Evaluation of this programme has resulted in positive feedback regarding the learning opportunities provided through active participation in the simulation. In contrast, mixed responses have been attained in relation to the observation component with the majority of students requesting a more active observer role. In addition, many students report uncertainty as to the technical and non-technical skills they are expected to look for whilst watching their peers.

**Fig. 1:**
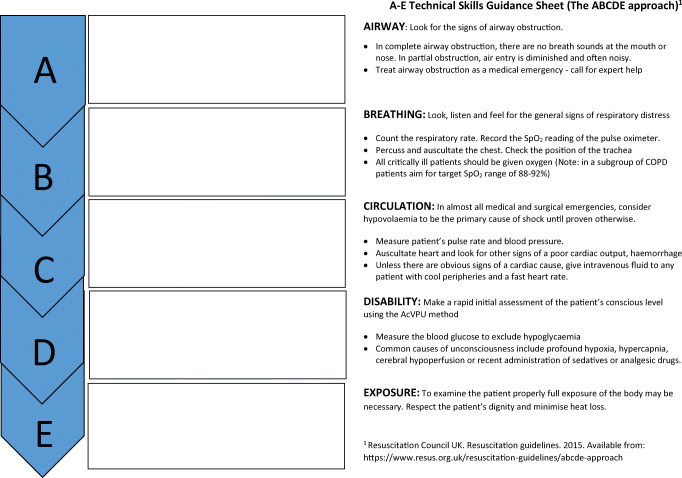
A–E technical skills guidance sheet

**Fig. 2:**
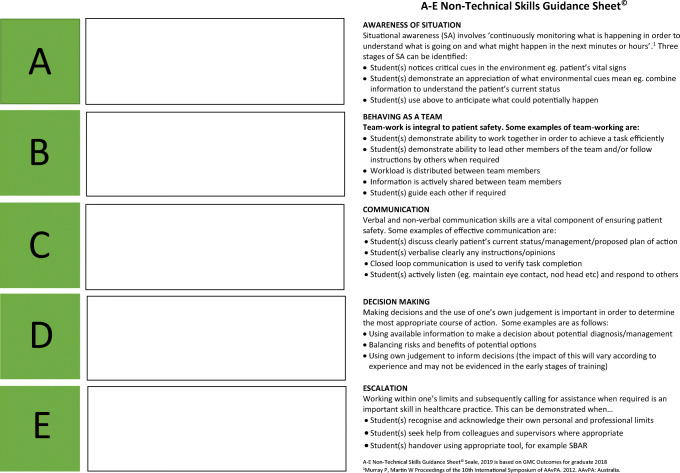
A–E non-technical skills guidance sheet

Previous research on simulation based education has advocated the use of observation tools in order to encourage directed as opposed to passive observation, a situation which has been reported to result in enhanced learner outcomes for the observer [1, 2]. In light of this, a unique observation tool was created which aims to foster a more active learning environment for students whilst they observe their peers. In response to student feedback, the tool also provides structured guidance on the technical and non-technical skills frequently demonstrated in clinical practice. The resultant observation aid consists of two A4 sheets of paper with a similar A–E labelled format. The ‘A–E technical skills guidance sheet’ (Fig. 1) is based upon the UK Resuscitation Council’s ABCDE clinical assessment of acutely unwell patients [3]. In contrast, the ‘A–E non-technical skills guidance sheet’ (Fig. 2) consists of a bespoke A to E abbreviation to categorise the skills students should be observing for during the simulation as follows: Awareness of situation, Behaving as a team, Communication, Decision-making, and Escalation. A brief description of each of these categories is provided for students to refer to if needed. All non-technical skills included in the sheet are based upon those detailed in the UK General Medical Council’s ‘Outcomes for Graduates’ [4]. Each sheet also provides space for students to record any instances in which the skills occur during the observed simulation.

The observation tool was subsequently trialled with year two KCL undergraduate medical students undergoing Hi-Fi SIM. During each half day simulation session, two medical students participated in one of four 10-min clinical scenarios involving the management of an acute clinical case focused at the level of skill expected from a year two KCL medical student. The remaining members of the group observed the scenario via a live video link. At the start of the simulation session, students were introduced to the observation tool which provided an explanation of its purpose and intended use. During each 10-min simulation, half of the observers were asked to complete an ‘A–E technical skills’ sheet and the other half an ‘A–E non-technical skills’ sheet. Students alternated between the two sheets throughout the half-day session to ensure everyone had the opportunity to complete each of the sheets at least once. Every scenario was followed by a 20-min debrief with two clinically qualified tutors during which time students were encouraged to discuss and reflect on any of the skills they identified from using the A–E sheets.

The observation tool was positively received by both the students and faculty involved in the simulation sessions. In particular, students commented that completing the sheets encouraged them to observe each simulation and enabled a more diverse debriefing which included both technical and non-technical factors. Tutors also identified a more in-depth analysis of the observed simulation during the debrief when compared with previous experiences without an observation tool. Following these encouraging reports, the intention is to use the observation tool as a teaching aid in all years of the KCL simulation-based undergraduate programme and to formally research whether their use translates into quantifiable differences in the acquisition of knowledge on technical and non-technical skills.
